# An environmental scan of one health preparedness and response: the case of the Covid-19 pandemic in Rwanda

**DOI:** 10.1186/s42522-021-00059-2

**Published:** 2022-01-16

**Authors:** Gloria Igihozo, Phaedra Henley, Arne Ruckert, Charles Karangwa, Richard Habimana, Rosine Manishimwe, Leandre Ishema, Hélène Carabin, Mary E. Wiktorowicz, Ronald Labonté

**Affiliations:** 1 Center for One Health, University of Global Health Equity, Kigali, Rwanda; 2 Global 1 Health Network, Ottawa, Canada; 3 School of Epidemiology and Public Health, University of Ottawa, Ottawa, Canada; 4 Rwanda Food and Drugs Authority, Kigali, Rwanda; 5 Faculty of Veterinary Medicine, Université de Montréal, Saint Hyacinthe, Canada; 6 Centre de Recherche en Santé Publique (CReSP), Montreal, Canada; 7 Groupe de Recherche en Épidémiologie des Zoonoses et Santé Publique (GREZOSP), Saint-Hyacinthe, Canada; 8 School of Health Policy and Management, York University, Toronto, Canada

**Keywords:** One health, COVID-19, Rwanda, Infectious disease management, Emergency preparedness, Pandemic response

## Abstract

**Background:**

Over the past decade, 70% of new and re-emerging infectious disease outbreaks in East Africa have originated from the Congo Basin where Rwanda is located. To respond to these increasing risks of disastrous outbreaks, the government began integrating One Health (OH) into its infectious disease response systems in 2011 to strengthen its preparedness and contain outbreaks. The strong performance of Rwanda in responding to the on-going COVID-19 pandemic makes it an excellent example to understand how the structure and principles of OH were applied during this unprecedented situation.

**Methods:**

A rapid environmental scan of published and grey literature was conducted between August and December 2020, to assess Rwanda’s OH structure and its response to the COVID-19 pandemic. In total, 132 documents including official government documents, published research, newspaper articles, and policies were analysed using thematic analysis.

**Results:**

Rwanda’s OH structure consists of multidisciplinary teams from sectors responsible for human, animal, and environmental health. The country has developed OH strategic plans and policies outlining its response to zoonotic infections, integrated OH into university curricula to develop a OH workforce, developed multidisciplinary rapid response teams, and created decentralized laboratories in the animal and human health sectors to strengthen surveillance. To address COVID-19, the country created a preparedness and response plan before its onset, and a multisectoral joint task force was set up to coordinate the response to the pandemic. By leveraging its OH structure, Rwanda was able to rapidly implement a OH-informed response to COVID-19.

**Conclusion:**

Rwanda’s integration of OH into its response systems to infectious diseases and to COVID-19 demonstrates the importance of applying OH principles into the governance of infectious diseases at all levels. Rwanda exemplifies how preparedness and response to outbreaks and pandemics can be strengthened through multisectoral collaboration mechanisms. We do expect limitations in our findings due to the rapid nature of our environmental scan meant to inform the COVID-19 policy response and would encourage a full situational analysis of OH in Rwanda’s Coronavirus response.

**Supplementary Information:**

The online version contains supplementary material available at 10.1186/s42522-021-00059-2.

## Introduction

Globally, 75% of new and re-emerging infectious diseases are of animal origin [1]. In low income regions such as sub-Saharan Africa, some estimates suggest that more than a quarter of disability-adjusted life years lost to infectious diseases are from zoonotic infections or emerging infections of animal origin [2]. East Africa is considered high-risk for emerging infections of animal origin; in the previous 5 years alone, it has experienced outbreaks of Ebola and Marburg, and most recently the SARS-CoV-2 pandemic [3, 4]. Further, over the past decade, 70% of emerging and re-emerging infectious disease outbreaks in East Africa have originated from the Congo Basin where Rwanda is located [5].

Rwanda has controlled and managed such past infections (COVID has yet to be suppressed), by creating measures to curb outbreaks of zoonoses. After realizing that illegal poaching and trading of wildlife meat were posing serious threats to the lives of humans and animals, the Rwanda Development Board implemented strict protection measures and discouraged illegal hunting and sale of wildlife meat by recruiting local communities and former poachers to become conservationists, and sharing 10% of revenues from national parks with the communities living around these parks [6]. Although these efforts have been successful in reducing risks associated with illegal hunting, Rwanda’s accelerated development, fast population growth and loss of biodiversity associated with increased human activity put the country at a heightened risk for the emergence of novel infectious agents of animal origins and zoonoses [7].

Global communities have recently recognized that solutions to global health threats require new ways of thinking [8–10]. One Health (OH), a multidisciplinary approach that calls on national, regional, and global collaborations to realize optimal health for humans, animals, and their environment, is gaining momentum [7]. OH calls on governments and non-state actors to prioritize inter-sectoral and multi-disciplinary collaboration, communication, and coordination to leverage financial and human resources to promote the health of people, animals, and the environment [10, 11].

**Fig. 1:**
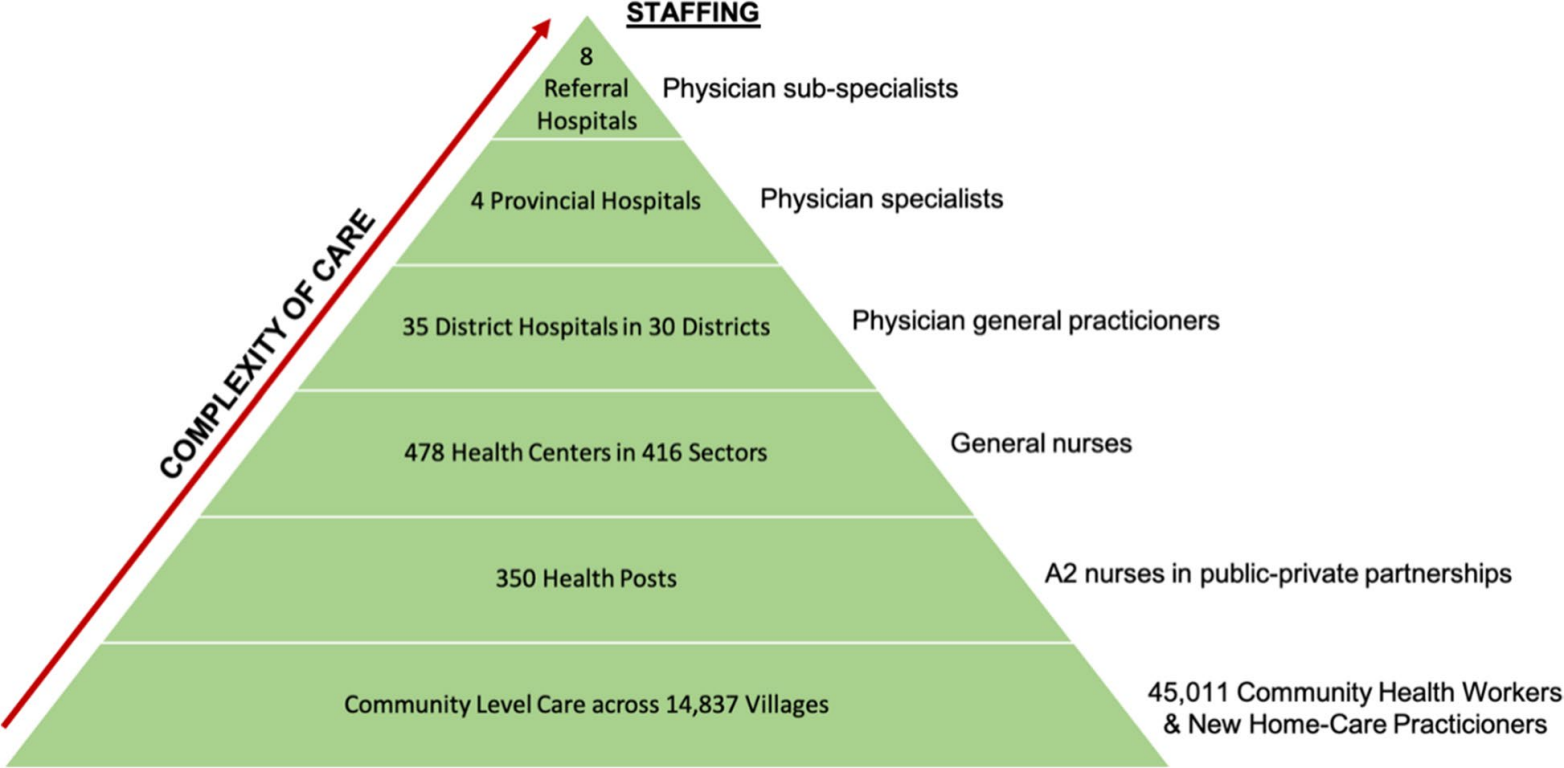
Rwanda’s decentralized healthcare system (adapted from Binagwaho et al., 2015) [15]

**Fig. 2:**
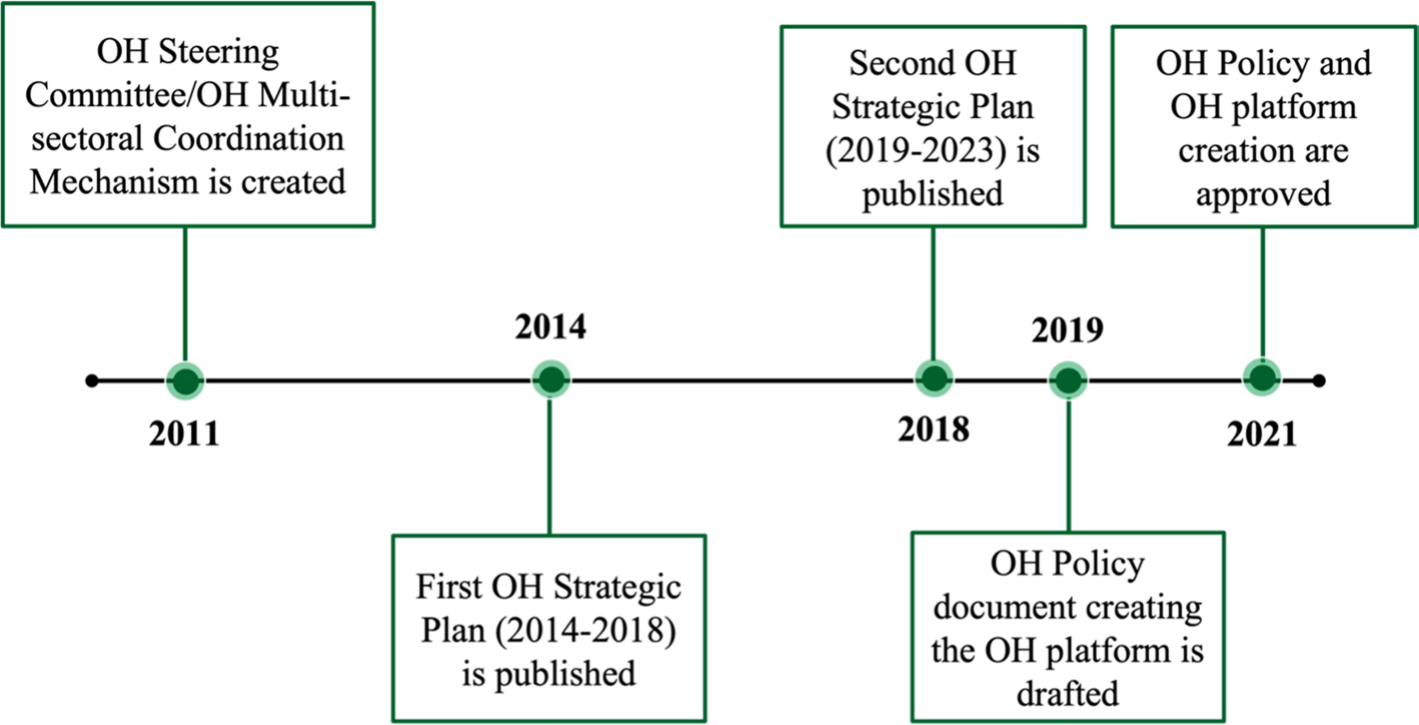
A timeline of the creation of Rwanda’s OH Governance Structure

Beginning in 2011, Rwanda has included OH in its governance systems and policies through the creation of a OH Steering Committee (OHSC), the development of two OH Strategic Plans, and the establishment of OH Policies [12–14]. Along with the decentralization of human (Fig. 1) and animal health services to the community level, Rwanda’s creation of a OH structure has prepared the country to address emerging health challenges Fig. 2.

Before the World Health Organization (WHO) officially declared COVID-19 a pandemic and before there was a confirmed case in-country, Rwanda had adopted an elimination approach [16, 17] to contain COVID-19 by screening passengers at all Points of Entry and opening a tracking and testing facility. The government created a national COVID-19 preparedness and response plan and set up committees, rapid response teams (RRTs), and task forces [18]. After its first confirmed case, Rwanda declared a nation-wide lockdown and closed its borders, the first country in Africa to do so [19]. Rwanda’s efforts to contain the COVID-19 pandemic have gained public recognition from other countries and health organizations [20].

Although the COVID-19 response in Rwanda has been extensive, how OH principles were integrated remains an open question. Our objective was to conduct an environmental scan to examine Rwanda’s OH structure and its role in the country’s response to COVID-19.

## Methods

For this study, OH principles are defined as having structures, policies, mechanisms, and platforms that facilitate collaborations and communications among various sectors and disciplines to address any health threat at the health of human-animal-environment interface.

A rapid environmental scan [21] of published and grey literature was conducted to assess how principles of OH are part of Rwanda’s governance response systems to zoonotic infections and COVID-19 (for detailed scan protocol, see Supplement 1). Through informal information gathering with OH stakeholders and online search, documents relevant to: (I) Rwanda’s OH governance structure, (II) Rwanda’s integration of OH into health systems; and (III) Rwanda’s response to COVID-19, were identified. A total of 132 documents were selected and obtained from official government institution websites and social media pages, google search engine, google scholar, and PubMed. Search terms included *“Rwanda (AND) COVID-19 (OR) Coronavirus,” “Rwanda (AND) COVID-19 (OR) Coronavirus (AND) One Health,”* and *“Rwanda (AND) One Health.”*

Documents were coded deductively using thematic content analysis in NVivo (NVivo 12). The codebook was collaboratively created in advance of data collection, informed by the Tripartite Guide for Addressing Zoonotic Diseases through OH in Countries [22] (for full Codebook, see Supplement 2).

## Results

Our results are presented in two sections: first, we describe Rwanda’s existing OH governance structure for zoonotic diseases (developed before the COVID-19 pandemic); and second, we analyze how this structure was invoked and OH principles applied in Rwanda’s response to COVID-19.

### Rwanda’s OH governance structure

The One Health Steering Committee (OHSC) - now known as the One Health Multisectoral Coordination Mechanism (OH-MCM) - created in 2011 is composed of different OH stakeholders, including the Ministry of Health, the Ministry of Agriculture and Animal Resources, and the Ministry of Environment. The OHSC was created to facilitate multidisciplinary and intersectoral collaborations in control and prevention of zoonotic diseases and other public health threats. Through the OHSC, Rwanda selected six zoonotic diseases to be prioritized (brucellosis, Human African Trypanosomiasis, viral hemorrhagic fevers, Highly Pathogenic Avian Influenza, rabies, Rift Valley Fever) of which preparedness and response plans were developed for the latter three.

The OHSC brought together stakeholders from the human, animal, and environmental health sectors to create a OH Strategic Plan in 2014 (OHSP I, 2014–2018) and again in 2018 (OHSP II, 2019–2023). Both strategic plans prioritize integrating OH in public health interventions and aim to: “(I) Promote and strengthen interdisciplinary collaboration and partnerships in OH approach, (II) Strengthen surveillance, early detection, rapid response, prevention, control of zoonosis, antimicrobial resistance and other public health threats, and (III) Build capacity and promote applied research at the human-animal-ecosystem interface” [12, 13].

**Fig. 3:**
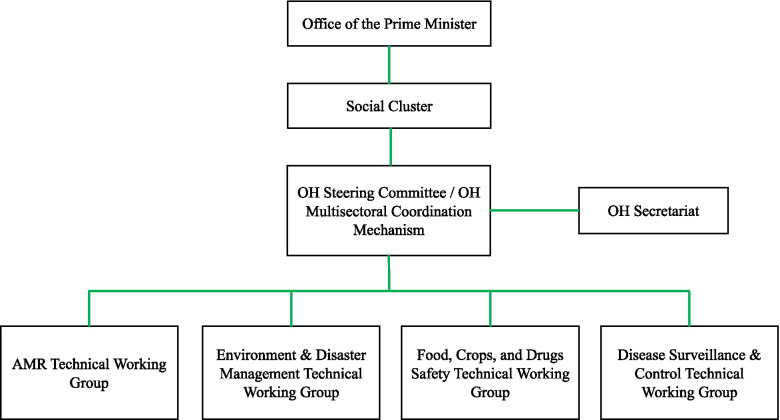
Rwanda’s OH governance framework adapted from the One Health Policy [14]

The OHSC developed a OH Policy in 2019 (approved in 2021) to create a OH platform (also known as the OH governance framework) and facilitate collaborations amongst sectors. The OH policy prioritizes objectives described in the OHSPs and outlines activities critical to implementation. The OH-MCM platform (Fig. 3) oversees the coordination and implementation of the OH policy. The OH-MCM previously supplemented the work of the OH Steering Committee and was “responsible for the overall governance [of OH strategies] including establishing strategies, prioritizing funding allocations, advocating and mobilizing resources for OH” [12, 13]. However, in the recent OH policy, the OH-MCM and OHSC have coalesced into one structure [14].

Rwanda’s OH strategy aligns with domestic and international legislations, policies, and codes relevant to a OH approach including the International Health Regulation (2005), the International Animal Health Organization Animal Health Code, and the Rwanda National Wildlife Policy. Specific recommendations from these three documents, such as setting up prevention measures at points of entries and streamlining collaboration and communication across all sectors responsible for human, animal, and environmental health were integrated into the strategic plans during their development. The OH strategic plans and policies seek to promote equity in decision-making by involving a range of relevant stakeholders (Supplement 3) in the development of OH plans and policies, decisions surrounding how funds are used, and encouraging all relevant OH sectors to collaborate across disciplinary, professional, and institutional boundaries. The documents outline the roles and responsibilities of each stakeholder in coordinating and implementing the goals and activities of each plan and policy. The OH platform, or OH governance framework, was established to coordinate and oversee the implementation of all OH Strategic Plan activities at the national level.

The office of the Prime Minister oversees the implementation and coordination of all activities in the OH Platform/OH governance framework (Fig. 3), assisted by a social cluster (a team of key ministries related to OH: Ministry of Health, Ministry of Agriculture and Animal Resources, Ministry in charge of Emergency Management, Ministry of Environment, and Ministry of Education) that provides policy guidance and approves action plans and reports. A OH Secretariat, composed of a program manager, three OH professionals and an administrator, was created to assist the OHSC/OH-MCM in the daily management of the OH Strategic Plan. Technical Working Groups provide expertise on different OH issues including antimicrobial resistance (AMR), zoonotic diseases and OH workforce development, and assist the OH-MCM by managing the implementation of thematic and technical activities of the OH Strategic Plan.

The OH platform recognizes the challenge of limited funding and uses a “common basket” (flexibility) approach where local and international partners and stakeholders willing to support activities will contribute funds to implement specific OH activities [12, 13]. The platform has.

secured a $50,000 seed grant from the Food and Agriculture Organization and a $250,000 donation from the Africa Development Bank to support the operationalization of the platform, to date.

Rwanda further developed its OH structure so it could respond to epidemics, epizootics, and environmental disasters. The country’s human and animal (wildlife and domestic) sectors are each equipped with laboratories with the capacity to diagnose a variety of infectious agents (Supplement 4), which are coordinated by a central lab; the human laboratories go up to biosafety level 4 while animal laboratories go up to biosafety level 2. All human health laboratories are coordinated by the National Reference Laboratory (NRL), which is decentralized to the district level and has created satellite laboratories to detect and alert the health system of cross-border infectious disease outbreaks. Animal Health laboratories, decentralized through district and satellite laboratories, are coordinated by the Rwanda Agriculture Board Central Laboratory and the Rubirizi National Veterinary Laboratory. These laboratories can detect a variety of pathogens using serological tests, molecular tests, and other techniques. While the animal health central laboratories have PCR capacity, satellite laboratories do not. Although each sector is responsible for its own monitoring and surveillance, both sectors occasionally consult one another on zoonotic diseases. Moreover, the human and animal laboratories work in silo; it is also unclear if they can detect the same zoonotic agents.

Rwanda’s OH approach aims to “empower and mobilize various experts and lay workers and establish a OH workforce to prepare, coordinate and manage epidemiological outbreaks of infectious, toxic or environmental health concern[s] or health events” [7]. OH has been integrated into curricula at the University of Rwanda’s undergraduate courses and in the medical and Master’s in Global Health Delivery program at the University of Global Health Equity. Both universities have also launched active Student OH Innovation Clubs to provide up to 800 students with OH competencies and encourage OH solutions. Further, the health sector strategic plan also requires that veterinarians, wildlife, and environmental experts be included in emergency management committees. During outbreaks, the government will also avail OH human resources by developing training programs and workshops to launch multidisciplinary Rapid Response Teams [5]. Lastly, in August 2021, Rwanda Biomedical Center- the implementing body of Rwanda’s Ministry of Health- created a OH Unit with the mandate to promote OH approaches in research, disease surveillance, and zoonotic and infectious disease management among others.

Rwanda’s OH policy prioritizes strengthening surveillance to allow for the detection, monitoring, and management of zoonotic diseases. The animal and human health sectors have surveillance reporting systems that the country continues to maintain and strengthen. The government, in 1998, created an Infectious Disease Surveillance Response (IDSR) system to “strengthen national public health surveillance and response systems” [23]. Additionally, Rwanda has also invested in electronic surveillance systems (electronic IDSR, the Immunization Monitoring Program, ACTive (IMPACT), and the Global Avian Influenza Network for Surveillance (GAINS), and the Influenza Sentinel Surveillance System) in public and private health facilities, to support early detection, reporting, tracing, and response to infectious diseases. Animal health surveillance, however, is not electronic and is based on annual disease detection studies of zoonoses including Avian Influenza, Rift Valley Fever, Foot and Mouth Disease, Newcastle Disease, and Ovine rinderpest among others. A multidisciplinary collaborative team of community health workers (CHWs), community-based animal health workers, healthcare facilities, park rangers, border agents, farmers, non-governmental organizations, and domestic (farm and companion) animal owners was created to monitor potential zoonotic disease outbreaks. The Rapid Response Teams created in response to Ebola and Avian Influenza outbreaks that occurred in neighbouring countries (Uganda and the Democratic Republic of the Congo) have continued to be used to address other OH and public health threats through surveillance, information sharing, and planning of risk reduction strategies.

### Role of OH in the Response to COVID-19

**Fig. 4:**
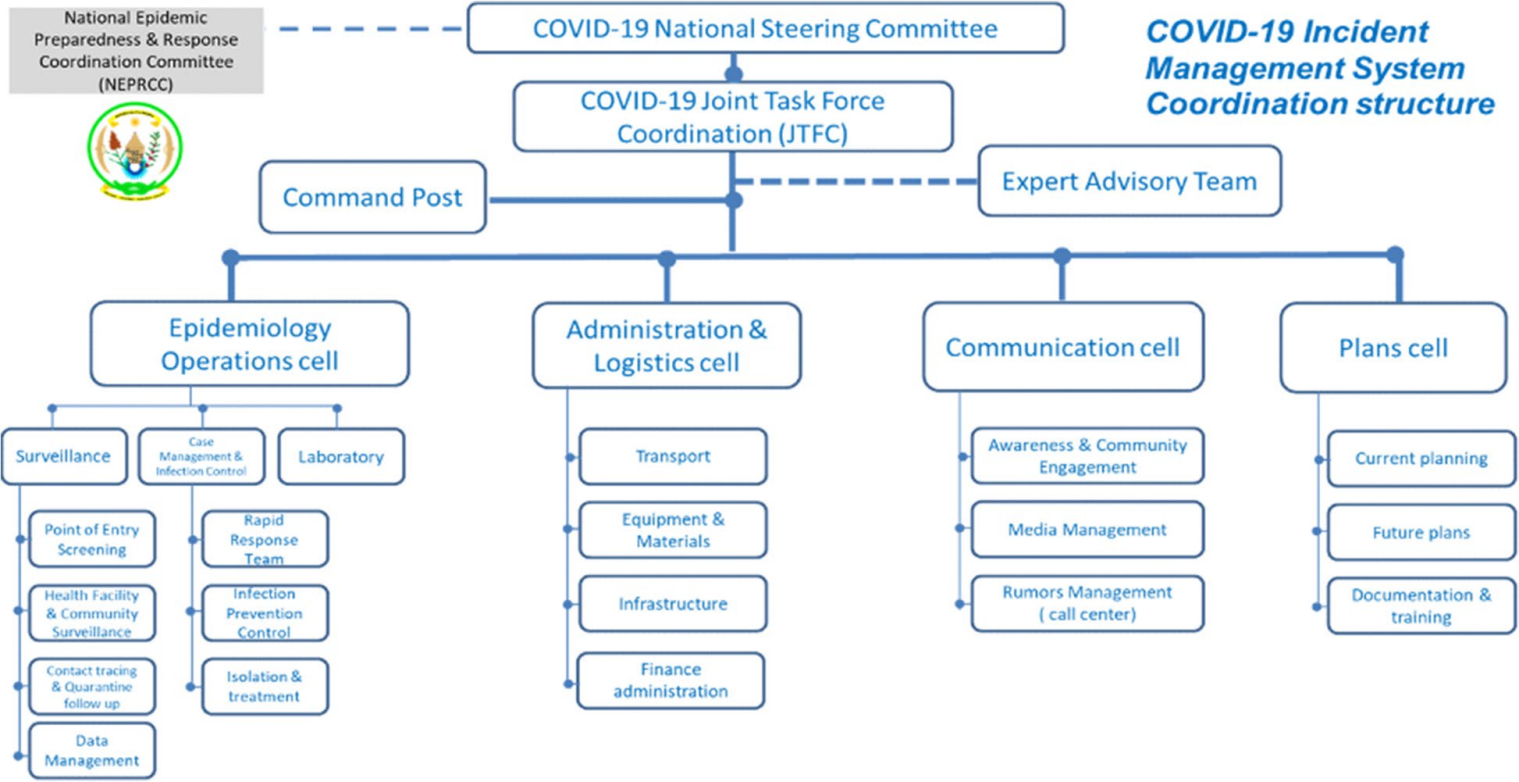
Rwanda’s COVID-19 multisectoral response structure [24]

Public health threats from neighbouring countries motivated Rwanda to increase surveillance and human resource capacity to detect and respond to potential outbreaks. Before COVID-19 was pronounced a pandemic, Rwanda set up a multisectoral response mechanism (Fig. 4). A national steering committee composed of different ministries, headed by the office of the Prime Minister, was created to assess, strengthen, and coordinate preparedness and response to COVID-19. This committee gathered different stakeholders including the Ministry of Health, the Ministry of Finance, the Ministry of Local Governance and the Rwanda National Police (RNP) to develop a National COVID-19 Preparedness and Response Plan that would “enhance the capacity to prevent, timely detect and effectively respond to a potential COVID-19 outbreak in Rwanda” [24]. The plan, operational from March to August 2020, outlines activities to manage and contain COVID-19 and specifies which institutions, ministries, and teams are responsible for each activity.

The committee established a multidisciplinary COVID-19 Joint Task Force and a command post to coordinate and implement the response to COVID-19 and the activities outlined in the preparedness and response plans. The task force included human and animal health professionals, both working together to monitor and address outbreaks in humans and animals. Veterinarians held decision-making power in the task force leadership team and worked alongside physicians to coordinate an equitable response. Drawing from their experiences in managing viral and infectious diseases such as Foot and Mouth Disease in animals, veterinarians and animal health professionals worked with human health doctors and public health professionals to develop effective prevention measures that could help limit the spread of COVID-19 in the community.

Further, rapid response teams created from a National Disaster Executive Committee, with representatives from nine government ministries including the human, animal, and environmental health sectors, were decentralized to district and community levels to evacuate confirmed COVID-19 cases. These teams worked with environmentalists who understood the role of the surrounding environment in infectious disease transmission to determine what to wear during evacuation and how to carry out decontamination. In its interventions, the government adopted a “decentralization strategy, where experts are deployed to different provinces to help districts to build their capacities [to respond to] COVID-19” [25]. Overall, more than 4000 professionals based in different sectors (security, immigration, veterinarians, physicians, and public health professionals) worked across different intervention areas including surveillance, contact tracing, testing, case management, psychological support, risk communication, and community engagement.

Using its own funds, loans and grants from donors, and public private partnerships, Rwanda mobilized resources and equipment to contain COVID-19. With these resources, the country strengthened the existing OH structure through opening ten more COVID-19 testing centers, creating a mobile laboratory, and increasing testing capacity fifteen-fold (from 1000 samples per day to 15,000 samples per day) between March and July 2020 [18]. By training CHWs, police officers, university students, veterinarians, and other professionals to be contact tracers, the government developed a contact tracing system that could trace 95% of diagnosed COVID-19 patient’s contacts [26]. The contact tracing center has multidisciplinary teams and a “close and coordinated response between different departments such as communications, mental health, police and immigration representatives as well as the RRT”, which has eased the ability to timely trace contacts and connect patients to appropriate care [27].

With the IDSR system and the sentinel surveillance system for respiratory diseases, the government closely monitored COVID-19 infections in all health facilities, points of entry, and at different community levels. The sentinel surveillance system closely monitors influenza outbreaks, the emergence of novel influenza viruses, and maps out types of outbreaks in sentinel sites which are mostly public health facilities [28]. Prior to the first confirmed case, the NRL had been equipped with technical and human resources to test for COVID-19. Between April and July 2020, satellite laboratories were opened in districts, focusing on hotspots, and more testing centers were opened in referral, district, and private hospitals. The government trained over 219 healthcare providers, 45,000+ CHWs, public health experts, and laboratory technicians on handling COVID-19 cases [29]. Twelve laboratory technicians from NRL were trained as trainers on COVID-19 sample collection and were used to train more laboratory technicians in district satellite laboratories country-wide [29]. In addition to human surveillance, the task force also initiated surveillance in animals by conducting COVID-19 testing in wild and companion animals that were considered susceptible to the infection such as gorillas, chimpanzees, cats, and dogs. Although no animals tested positive, data from this testing informed regulations to protect these animals from exposure to the virus. Furthermore, in June 2021, the taskforce worked with veterinarians in the Rwanda Agricultural Board to pilot COVID-19 testing using sniffer dogs; the dogs are believed to have a sensitivity of 94%, which is close to that of a PCR test [30, 31].

COVID-19 containment measures were also extended to animal and environmental health sectors. Realizing that gorillas and chimpanzees are susceptible to human respiratory pathogens, three national parks were closed to avoid cross-species contamination. When these parks were reopened, tourists were required to have a negative COVID-19 test 48 h prior to visiting. Additionally, Rwanda Environment Management Authority and the COVID task force recognized that mishandling of used face masks may result in pollution of water, soil, air and increase COVID-19 transmission and spill over risks, and established guidelines on collecting and safely disposing of used face masks.

To increase COVID-19 awareness among the public, a national communication plan was developed. Various communication channels (social media, telephone messages, television, radio, billboards, and posters) were used to educate community members on COVID-19 symptoms, transmission and prevention. The government worked with Rwanda National Police to disseminate this information to densely populated neighborhoods, high-risk zones, and geographically hard-to-reach communities, including remote villages, using drones. Daily COVID-19 updates (number of new cases, deaths, and recoveries) are posted on the Ministry of Health’s social media accounts and on Rwanda’s national radio and television stations.

## Discussion

While the importance of applying OH principles in the preparation, response and management of zoonotic diseases has been widely acknowledged globally, the operationalization of OH remains limited in many countries and regions of the world [32]. The pandemic has exposed the urgent need to revisit disaster preparedness and evaluate public health responses to zoonotic diseases through the lens of OH [33]. Rwanda presents a positive example of how progress in OH preparedness can translate into effective zoonotic disease management, providing important lessons for other countries and regions of the world. It has limited the cumulative death count from SARS-CoV2 to about 1200 as of September 2021, and the cumulative confirmed deaths per million people to around 29, as compared to 1000 in Germany (widely considered a strong pandemic performer), and around 2000 in the case of the United States and Brazil (widely considered poor pandemic performers) [34].

Several factors enabled Rwanda to operationalize the OH approach effectively. The country has social and political stability at all levels of society. Coupled with political willingness and community support, this has enabled Rwanda to bring human, animal, and environmental health sectors together. Across government sectors, there is “political awareness on OH, created in part by President Kagame’s highlighting of OH in a key meeting on health security” [13]. Additionally, an extensive network of more than 45,000 CHWs, over 30 RRTs, 1000 community-based animal health workers, and international partnerships equip Rwanda with the human resource capacity to advance OH interventions. Further, Rwanda has human and animal health systems that are decentralized to communities and electronic surveillance systems for both human and animal health.

The human and animal sectors that are well-equipped laboratories that monitor and respond to potential epidemics, even though interdisciplinary engagement and collaboration between human and animal health laboratories remains limited. Integration of the surveillance systems has not been achieved yet, nor has effective exchange in surveillance data. Surveillance of infectious agents still occurs in silos in the field instead of being planned as an integrated system. This confirms the challenges of operationalizing the OH approach in the field. However, efforts towards this goal continue with “key ministries related to OH [having] already coalesced to form a ministerial ‘Social Cluster’ which meets monthly, with the goal of ensuring that there is little competition for resources between ministries and that shared issues are addressed collectively.” [7]. Lastly, experience with previous zoonotic disease outbreaks has contributed to Rwanda’s drive to strengthen OH. Therefore, Rapid Response Teams previously developed in response to zoonotic outbreaks, including Ebola, Rift Valley Fever and Avian Influenza, in neighbouring countries are used to address other OH problems [7].

Although Rwanda has advanced OH through policies, strategies, and structures that promote collaborations across sectors and disciplines, barriers remain in place limiting its effectiveness. Financial and human resource constraints are major setbacks, as “funding is still a problem in nearly all institutions which cripples ability to carry out joint response activities. This ultimately affects staff preparedness and readiness to respond to emergency threats” [7]. The country continues to face a shortage of human, animal, and environmental health professionals, as well as experts trained in OH. This challenge is not unique to Rwanda, but rather is a widespread problem across Africa, despite recent efforts to build OH capacity across the continent [35]. In addition, existing OH platforms are faced with ineffective communication strategies and a lack of accountability, which has resulted in many ministries continuing to work in silos. Rwanda has no One Health surveillance strategy including interdisciplinary laboratories or sample collection, making efficient early warning surveillance and response systems an ongoing challenge.

Finally, although situating the operationalization of the OH Platform and OH Secretariat within the Prime Minister’s office and ensuring support from a social cluster of key ministers reflects OH as a national priority, the OHSC has no formal positioning in government structure. Instead, it sits independently of other government institutions, and so its members “don’t have or feel the need to champion OH within their own institutions” [14]. As such, there is considerable room for improvement to further strengthen Rwanda’s OH structure, break up existing silos that remain entrenched in certain corners of Rwanda’s health governance structure, and create further momentum on the path towards OH implementation.

## Conclusion

The COVID-19 pandemic has reinforced the notion that effective management of zoonotic diseases can best be achieved through the integration of OH principles into a countries’ governance structure. Our environmental scan documented the extent to which OH principles have informed Rwanda’s COVID-19 policy response and highlighted remaining weaknesses that could be overcome to further strengthen the application of OH principles. Indeed, Rwanda’s experience presents important lessons for other countries, not just in Africa, and suggests that OH, as a well-recognized concept that has remained at the fringe of most operational health policies for far too long, must become a central aspect of infectious disease management and governance everywhere.

## Supplementary Information

**Additional file 1.**

**Additional file 2.**

**Additional file 3.**

**Additional file 4.**

## Data Availability

The datasets used and analyzed during the current study are available from the corresponding author on reasonable request.

## Abbreviations

CHWs: Community Health Workers
COVID-19: Coronavirus Disease of 2019
GAINS: Global Avian Influenza Network for Surveillance
IDSR/e-IDSR: Integrated Disease Surveillance Response
IMPACT: Immunization Monitoring Program, ACTive
NRL: National Reference Laboratory
OH: One Health
OH-MCM: One Health Multisectoral Coordination Mechanism
OHSC: One Health Steering Committee
OHSP: One Health Strategic Plan RRT Rapid Response Team
RNP: Rwanda National Police
SARS-Cov-2: Severe Acute Respiratory Syndrome Coronavirus 2
WHO: World Health Organization
