# Supplementary material for: An environmental scan of one health preparedness and response: the case of the Covid-19 pandemic in Rwanda
Source: One Health Outlook. 2022 Jan 16;4:2. doi: 10.1186/s42522-021-00059-2 (PMC8761094; doi:10.1186/s42522-021-00059-2)
Supplement: Supplementary file 1 — Additional file 1. [file 42522_2021_59_MOESM1_ESM.docx]

Supplement 1

**Research Protocol on Conducting an Environmental Scan of Country Level Preparedness and Policy Response to the Novel Coronavirus (Covid-19) and Similar Zoonotic Disease Threats**

**Background**

The proposed environmental scan is part of a Canadian Institutes of Health (CIHR) funded study on how the governance of infectious diseases can be improved by applying principles of One Health (OH) and considering equity consideration in the policy response. The recent Coronavirus outbreak (COVID-19) has reinforced that the OH approach to preventing or reducing zoonotic disease risk is at the forefront of global health challenges. Practical implementation of the OH approach to the governance of IDs has proven challenging. There are concerns that even those international organizations (WHO, Food and Agricultural Organization (FAO), and the World Organization for Animal Health (OIE) currently leading the promotion of the OH approach may be lacking in implementation capacity during emergency situations; and that many countries are lacking effective OH governance systems at the national level. However, health issues at the human-animal-environment interface cannot be effectively addressed by one sector alone. Collaboration across all sectors and disciplines responsible for health is required to address zoonotic diseases and other shared health threats at this interface. One Health describes a collaborative, multidisciplinary, and multisectoral approach that can address urgent, ongoing, or potential health threats at the human-animal-environment interface at subnational, national, global, and regional levels. This approach also includes ensuring balance and equity among all the relevant sectors and disciplines in the policy response.

The COVID-19 outbreak presents an opportunity to establish a real-time monitoring infrastructure to study and promote the embedding of OH principles within the national and global governance of infectious diseases (IDs). Employing rapid environmental scan methodology and building on already existing research collaborations, we will produce immediate results focused on the coordination and response systems that can feed into better global governance of COVID-19. The main objective of our

proposal is to improve evidence-based decision-making in the public health response to COVID-

19, and enhance international collaborative efforts to mitigate its spread using a OH approach. The environmental scan portion of the lager study will probe the extent to which OH principles are part of existing governance response systems in four countries (Brazil, Ecuador, Mexico, and Rwanda), and aims to provide recommendations for how pandemic preparedness can be improved through better integrating OH principles into governance systems.

Since equity considerations are central to the OH approach, we want to include another second dimension in the scan: the extent to which vulnerable populations are identified and supported through specific and targeted policy interventions in the Covid-19 response. There will be three parts to this scan expansion. The first part would describe and document (to the extent possible) the equity dimensions of the Copvid-19 outbreak in the case countries (i.e. who faces the greatest risk and why?). The second part would be an assessment of the equity dimensions of the country’s Covid-19 responses in terms of policy, funding, public communication, and new programming (i.e. how well does it account for fairness and ‘vertical justice’ in who receives public assistance?). The third part is more forward looking: What policy domains need fundamental rebooting to avoid returning to a pre-covid normal that was already highly inequitable, and produced the widely documented inequities in Covid-19 contraction and mortality?

**Procedural Steps in the Environmental Scan:**

**Step 1:** Draw on experience to determine leadership and capacity for the project

A coordinator or team member must be designated to champion the entire environmental scan process from development to dissemination. This person will be the point of contact with the research team at the University of Ottawa which is providing logistical support for the scan. The contact person at the University of Ottawa is Dr. Arne Ruckert ([aruckert@uottawa.ca](mailto:aruckert@uottawa.ca)), please feel free to contact Arne at any point if you have any questions about the environmental scan.

**Step2**: Establish a Focal Area and Purpose of the Environmental Scan

It is critical to specify a purpose for the environmental scan to anchor the process and focus the organization’s limited time, energy, and resources. The aim of our environmental scan is to draw on recent experiences that your country has had with emerging or re-emerging Infectious Diseases (IDs), and especially SARS-COV2 and Covid-19, to address the following questions:

- How is OH conceptualized (known, defined, understood) within your country?
- What are existing OH capacities and governance mechanisms to address emerging or re-emerging infectious diseases in your country?
- How did OH principles influence policy responses during recent infectious disease outbreaks (including the current COVID-19 outbreak) in your country?
- What organizational and policy changes could better prepare the country to respond to future emerging and re-emerging infectious disease outbreaks based on OH principles (please provide specific policy recommendations)?
- To what extent do inequities in the distribution of Covid-19 disease burden exist, and are documented by your government
- To what extent are equity issue communicated in the official Covid-19 policy response, and how?
- What are equity dimensions of the fiscal policy response (emergency funding for poor; business bailouts)
- What policies might be needed to ensure more equitable access to social determinants of health going forward

**Step 3:** Create and Adhere to Timeline and Incremental Goals

The environmental scan has a 6 month timeline from start to finish since it is part of a rapid Coronavirus response grant provided by the Canadian Institutes for Health Research (CIHR), and consists of the following incremental steps:

May 2020: Establish goals of scan, approach research collaborators and knowledge users; identify relevant sources of information; approach government departments with relevance to One Health governance to identify additional resources that might not be available online (see Step 4 for more details)

June 2020: Start search for relevant documents to be coded and build Zotero database (Arne Ruckert to connect you to the project’s Zotero database and to provide training on Zotero use, if necessary in May 2020)

July-August 2020: Develop coding scheme and analyze research findings using thematic content analysis (Ottawa U to provide online training if necessary in June 2020, see more details under Step 6)

September-October 2020: Draft research report (4000-5000 words); receive feedback from Ottawa U research team on draft report; revise research report; develop Policy Brief (1000 words) in collaboration with Ottawa U

**Step 4.** Determine Information to be collected for Environmental Scan

Brainstorm all topics and resources that could inform the environmental scan. All desired information may not be available, but include everything that, ideally, should be part of the scan. Casting a wide net and finding that information is unavailable is better than risking missing something important. Unlike Step 2, the list of items in this step will be dynamic, changing as opportunities to engage stakeholders develop and new resources are discovered. Given the short turn-around time of the research project, we initially expected that the scan would be performed only using document analysis methodology. However, if you feel that additional key informant interviews (KIIs) are necessary, and you are able to perform those within the tight timeline of the research project, feel free to conduct additional KIIs to collect information.

We recommend the following initial steps to identify relevant information, but feel free to adjust to your unique national context:

1. Approach other experts in the field of One Health and familiar with governance landscape to ask about useful resources to consult (these are not supposed to be formal interviews but rather informal conversations about where to look for information, and as such should not require ethics approval for research with human subjects)
2. Based on these informal conversations, identify resources (government documents on one health, NGO documents, WHO evaluation of capacity to address International Health Regulations, other international agencies assessment of pandemic preparedness, etc.)
3. Conduct library (grey text database) and online research (Google or other more targeted search engines) for additional relevant documents (please systematically record how the search was conducted, date, search terms and how they were combined, databases, search engines, etc.)
4. If deemed necessary, identify potential key informants for semi-structured interviews based on information provide under 1-3 above (some research sites have suggested they want to conduct interviews; please be aware that this might slow down the scan and should not compromise the project’s timeline)

**Step 5:** Identify and Engage Stakeholders:

Stakeholders, and their willingness to participate in the environmental scan, are the key to success. Create a diverse, iterative table of people or organizations that have information on each topic named in Step 4. Stakeholders may expand the original list of topics/ questions by recommending or connecting project staff members to other stakeholders (i.e., snowball approach), and might also provide access to additional documents that might not be available online.

**Step 6**: Analyze all collected data by conducting thematic content analysis using NVivo12 or similar qualitative research software (coding tree to be developed deductively based on the Tripartite Addressing Zoonotic Disease Guide and reviewed by all project teams through online teleconference in June 2020). As part of the analysis we are particularly interested in assessing the existences of One Health infrastructure and policies. Using the Tripartite Guide to Addressing Zoonotic Diseases through One Health in Countries, we identified the following Multisectoral Coordination Mechanisms (MCM) as a basis for analysis of the existing OH infrastructure:

• sector-specific structures and infrastructures, responsibilities, programmes, and activities related to zoonotic diseases, including sector specific needs and priorities

• international and regional obligations (such as capacity to abide by International Health Regulations)

• collaborative, multidisciplinary, multisectoral, and/or One Health-specific activities and programmes within or among the relevant sectors (for example a standing One Health parliamentary committee)

• activities in partnership with central or subnational government, universities, nongovernmental organizations, industry, or others, in sectors other than those of human health, animal health and the environment, and at central or subnational levels

• frameworks, protocols, plans, and/or strategies relating to zoonotic diseases created by sectors or collaboratively among sectors

• human and financial resources available for implementation of One Health governance mechanisms and principles (sufficiency)

• enablers to expanded OH collaboration

• barriers to expanded OH collaboration

But the specific coding tree and guidelines for thematic analysis will be developed collaboratively across all research sites in a teleconference in June 2020.

**Step 7:** Disseminate Results and Conclusions to Key Stakeholders

After we have finalized the report, we will develop a Policy Brief based on the research findings in collaboration with the research teams to share with key stakeholders (1000 words). Finally, we will make the results of the environmental scan widely available, including to the funding agency, the organization’s leadership, and those who participated in the process.

**Resources Relevant to all Country Sites:**

A Tripartite Guide to Addressing Zoonotic Diseases in Countries (OIE, FAO, and WHO 2019)

https://www.oie.int/fileadmin/Home/eng/Media_Center/docs/EN_TripartiteZoonosesGuide_webversion.pdf

This guide is crucial as it very clearly sets out expectations of what effective One Health governance structures would look like. It highlights what mechanisms and structures might exist, or would be desirable to put in place. I would recommend this as a starting point for every researcher to read, so as to understand what an effective one health governance set-up would look like, and to understand what information needs to be sought out.

General guidelines for environmental scans

<https://www.cdc.gov/pcd/issues/2016/16_0165.htm>

This is a very useful practical resource that describes the steps necessary to conduct an environmental scan.

Example of environmental scan applied in a health settings:

<https://www.ncbi.nlm.nih.gov/pubmed/28494638>

Feel free to contact Arne Ruckert at [aruckert@uottawa.ca](mailto:aruckert@uottawa.ca) at any point regarding any questions you might have!
