# Supplementary material for: An environmental scan of one health preparedness and response: the case of the Covid-19 pandemic in Rwanda
Source: One Health Outlook. 2022 Jan 16;4:2. doi: 10.1186/s42522-021-00059-2 (PMC8761094; doi:10.1186/s42522-021-00059-2)
Supplement: Supplementary file 2 — Additional file 2. [file 42522_2021_59_MOESM2_ESM.docx]

Supplement 2

**Code Book - Environmental Scan of One Health Preparedness and Response Capacity for Zoonotic Diseases**

| **Dimension** | **Node** | **Subnode** | **Thematic Inclusions Criteria** |
| --- | --- | --- | --- |
| **One Health**  **Equity** | Definition |  | How is health equity defined? What are its key dimensions? |
|  | Disaggregated data |  | What disaggregated data, analysis, and use of information exists, to ensure that responses are effective and differentiated, according to the needs of diverse populations (e.g. disaggregated data by sex and gender, race, income level, work status, etc.)?  Are inequities in the distribution of disease risk identified?  What groups are singled out as particularly vulnerable? |
|  | Equity dimension of fiscal policy | Business bailouts | How are equity considerations integrated into business bailouts?  Who receives most of the emergency funding to the business community, and is there a focus on small businesses? |
|  |  | Emergency program spending | What social safety net has been put in place?  Are equity considerations part of emergency program spending? For example, are there specific funding envelopes or programs for vulnerable populations (e.g. homeless, indigenous, racialized and LGTQ+ communities, informal workers, etc.) |
|  |  | Social Determinants of Health | How is emergency and recovery spending impacting various social determinants of health pathways, such as housing, education, and income security? |
|  | Gender |  | To what extent is there an attempt to explain and interpret the gender dynamics of COVID-19 to guide country responses, including conducting surveillance and specific data reviews on testing outcomes, transmission rates (including health workers’ infections), morbidity and mortality, hospitalization rates, access to health services, and risk factors?  Are there measures implemented to specifically address women’s dual burden and increased exposure and risk to COVID-19 and unfavorable mental health outcomes as key frontline healthcare workers and care providers, including improved access to information and personal protection equipment and promotion of flexible work arrangements.  Are there specific interventions to prevent and address gender-based violence including sexual violence and exploitation, especially under stay-at-home policies?  Is there an acknowledgment of the disproporniate impact of the economic collapse related to Covid-19 on women (it is a shecessions not recession!)? |
|  | Human Rights | Policy Implementation | How are human rights principles invoked in policy documents?  Is access provided to testing related to COVID-19, without discrimination on any grounds?  Is there availability of treatment, without discrimination on any grounds? |
|  |  | Political and Social Rights | What restrictive measures (e.g. lock-downs) are implemented, and are those in accordance with Article 3 of the International Health Regulations (2005), which require respect of the dignity, human rights, and fundamental freedoms of persons?  Are restrictive measures that limit civil and political rights monitored to ensure they are strictly necessary to respond to COVID-19 and are aligned with the principles of reasonability, proportionality, and duration?  Is information broadly available in multiple languages (including for indigenous communities), and accessible to people with vision, hearing, learning, and other disabilities? |
|  |  | Right to Health | The right to health is closely related to and dependent upon the realization of other human rights, including the rights to food, housing, work, education, non-discrimination, access to information, and the freedom of movement. These and other rights and freedoms are integral components of the right to health.  Are special measures related to other human rights implemented, for example, rights related to housing (e.g., suspension of mortgages, rent payment, and safe and adequate shelter for homeless), food (e.g., distribution and access to food for people in situations of vulnerability), and education (e.g., access to computers and internet and food in lieu of school meals)? |
|  | Identification of vulnerable populations |  | Are vulnerable populations identified? And if so, what population groups are identified as vulnerable? |
|  | Social determinants of pandemic outcomes |  | Are social determinants discussed in the context of pandemic preparedness and response?  What specific social determinants are mentioned (housing, employment status, income, gender, health systems, etc.), and how? |
|  | Targeted Interventions |  | Are there targeted and adapted actions to enable access to COVID-19 health services for populations in situations of vulnerability? This can include, amongst others: emergency policies, plans, and guidelines to address financial barriers to COVID-19 health services, including, for example, avoiding out-of-pocket payments, user fees, and catastrophic expenditure; and decentralized testing and care facilities to provide access to geographically disbursed populations and to facilitate transportation to testing for those with suspected cases |
|  |  |  |  |
| **One Health Governance** | Barriers to One Health |  | Are there specific barriers mentioned to the implementation of One Health principles? |
|  | Enablers of One Health |  | Are there specific enablers mentioned for the implementation of One Health principles? |
|  | One Health Infrastructure | Communication and Education | Are there strategies or plans for information sharing, crisis communication, and health education on the importance of OH?  Is communication transparent and accessible?  Is there an early warning system for zoonotic disease outbreaks?  Is there a real time notification system?  Are there regular risk and situational assessments? |
|  |  | Human Resources | Are there adequate human resources working on OH implementation?  Is there adequate funding to ensure HR availability? |
|  |  | Laboratories | Is there adequate laboratory capacity to identify zoonotic diseases?  Are there sample testing procedures in place?  Is there environmental testing? |
|  |  | Multi-sectoral Coordination Mechanisms (MCMs) | Are there dedicated OH committees at federal and provincial levels, or similar multi-sectoral coordination mechanisms?  Do these committees or similar MCMs meet on a regular basis?  Is there wide-spread participation by a variety of cross-sectoral stakeholders in MCMs?  Are there clear procedural guidelines for activation of MCMs during health emergencies? |
|  |  | OH Legislation | Are the International Health Regulations (IHRs) or other regional health obligations mentioned in documents? And if so, how do they contribute to OH preparedness and response?  What are existing domestic legislative and regulatory elements of OH preparedness and response? |
|  |  | Public Private OH Partnerships (PPPs) | Are there any PPPs in OH implementation?  What is the role of private partners in such PPPs? |
|  |  | Sector-specific OH Structures, Programs, Plans and Activities | Are the any OH structures, programs, plans and activities that are limited to specific sectors?  What are the main sectors where such structures, programs, plans and activities exist, with what specific policy goals?  Is Personal Protective Equipment (PPE) widely available in relevant sectors of economy? |
|  |  | Subnational OH Frameworks, Programs, Plans, and Initiatives | What subnational OH frameworks, programs, plans, and initiatives exist, with what specific policy goals?  How do these related to national initiatives? |
|  |  | Surveillance | What programs of epidemiologic surveillance in humans, exist?  What programs of epidemiologic surveillance in animals exist  Are there any joint or integrated surveillance programs for Covid-19, or any other zoonotic disease?  Are there joint (OH) field investigation teams, and do such teams receive training?  Do animal breeders participate in any OH surveillance systems? |
|  |  | Tracing Capacity | What capacity exists regarding contact tracing of zoonotic diseases? Was this ramped up in response to Covid-19? |
|  | OH Preparedness and Response Plans | Existing Frameworks, Protocols, and Plans | What are existing OH frameworks, protocols, and plans? |
|  |  | Participation of Sectoral Stakeholders in Development | What sectoral actors participate in the development of existing OH frameworks, protocols, and plans? |
|  |  | Resourcing of existing OH frameworks, protocols, and plans? | Do existing OH frameworks, protocols, and plans receive the funding required to work properly? |
|  |  | Roles and Responsibilities or Stakeholders | What are the roles and responsibilities or various stakeholders? Are they properly defined and set out clearly? |
| **One Health Prevention** | Antimicrobial resistance |  | How is antimicrobial resistance discussed in relation to zoonotic disease risk and response? |
|  | Biosafety and Biosecurity |  | What are biological/infectious hazardous waste management protocols  How are dead bodies dealt with? |
|  | Food safety | Animal identification and traceability | Are animal and meat products traceable to the source? |
|  |  | Regulation and inspection | What does the regulation, authorization and inspection of establishments for production, processing and distribution of food of animal origin look like? |
|  | Disinfection of public areas |  | Are there specific protocols for disinfection of public areas? |
|  | Environmental risk factors |  | Are environmental risk factors discussed, including air pollution, chemicals in the soil, habitat loss, introduction of invasive species, land use changes, climate change, deforestation and the impact of extractive industries such as mining; and how these might impact zoonotic disease transmission risk? |
|  | Immunization |  | How is immunization discussed in relation to zoonotic disease preparedness and response? |
