# Supplementary material for: An environmental scan of one health preparedness and response: the case of the Covid-19 pandemic in Rwanda
Source: One Health Outlook. 2022 Jan 16;4:2. doi: 10.1186/s42522-021-00059-2 (PMC8761094; doi:10.1186/s42522-021-00059-2)
Supplement: Supplementary file 3 — Additional file 3. [file 42522_2021_59_MOESM3_ESM.docx]

Supplement 3

**List of Rwanda’s One Health Stakeholders**

1. Ministry of Health
2. Rwanda Biomedical Center
3. Rwanda Agricultural Board
4. Ministry of Health and Animal Resources
5. Ministry of Environment
6. Rwanda Environment and Management Authority
7. Rwanda Development Board
8. Office of the Prime Minister
9. Ministry in charge of Finance and Economic Planning
10. Ministry in charge of ICT and Innovation
11. Ministry in Charge of Emergency Management
12. World Health Organization
13. Ministry of Education
14. University of Rwanda
15. University of Global Health Equity
16. Africa One Health University Network (AFROHUN), formerly OHCEA
17. CDC
18. USAID
19. Food and Agriculture Organization of the United Nations (FAO)
20. Rwanda Veterinary Association and Council
21. Rwanda Medical and Dental Council
22. Rwanda Food and Drug Authority
23. Rwanda National Police
24. Ihorere Munyarwanda Organization (IMRO)
25. Health Solutions for Africa
26. Sight and Life Foundation
27. World Food Programme (WFP)
