# Supplementary material for: An environmental scan of one health preparedness and response: the case of the Covid-19 pandemic in Rwanda
Source: One Health Outlook. 2022 Jan 16;4:2. doi: 10.1186/s42522-021-00059-2 (PMC8761094; doi:10.1186/s42522-021-00059-2)
Supplement: Supplementary file 4 — Additional file 4. [file 42522_2021_59_MOESM4_ESM.docx]

Supplement 4

**Pathogens and Diseases Detected in Human and Animal Laboratories in Rwanda**

*Please note: This list is not exhaustive.*

Avian influenza

Borrelia

Brucella

Canine parvovirus

Chikungunya virus

COVID-19

Coxiella burnetii

Dengue virus

Ebola Virus Disease

E.coli

Fungal, viral, and bacterial meningitis

Human Immunodeficiency Viruses (HIV)

Human Hepatitis B and C

Human Herpes Viruses

Human Papillomavirus (HPV)

Influenza B virus

Leptospira

Malaria

MDX Cancer markers for breast, lung, colon, blood, JAK2 mutation, BRAF, etc.

Measles

Microfilaria

Multi-Drug Resistance (MDR) Tuberculosis

Mycobacterium tuberculosis

Neisseria gonorrhoeae

Newcastle virus

Rabies

Respiratory viral and bacterial pathogens

Rickettsia

Rift Valley Fever (RVF)

Rubella

S. pneumonia

Salmonella

Shigella

Streptococcus

Syphilis

Tropical fevers Core and Africa multiplex

Trypanosoma

Viral gastroenteritis multiplex

Yersinia

Zika
